# Comparison Between China and Brazil in the Two Waves of COVID-19 Prevention and Control

**DOI:** 10.1007/s44197-022-00036-6

**Published:** 2022-03-30

**Authors:** Meiheng Liu, Leiyu Shi, Haiqian Chen, Xiaohan Wang, Manfei Yang, Jun Jiao, Junyan Yang, Gang Sun

**Affiliations:** 1 Department of Health Management, School of Health Management, Southern Medical University, Guangzhou, Guangdong 510515 People’s Republic of China; 2 Department of Health Policy and Management, Bloomberg School of Public Health, Johns Hopkins University, Baltimore, MD 21205 USA

**Keywords:** COVID-19, Containment strategy, Mitigation strategy, Meteorological factor, Policy evaluation

## Abstract

**Objective:**

This study analyzes the effectiveness of COVID-19 prevention and control in China and Brazil from the perspectives of policy and meteorological conditions, and provides experience for epidemic prevention and control.

**Methods:**

This study collects data on meteorological conditions, vaccination and mutant strains in the two countries to analyze the reasons for the differences in epidemic status between the two countries and extracts public data on COVID-19 through various official websites, summarizes the prevention and control policies implemented by the two countries, and evaluates their effectiveness.

**Results:**

As of August 12, 2021, the total number of COVID-19 cases and the daily number of new COVID-19 cases in China have been growing steadily, showing remarkable results in epidemic control. The total number of confirmed cases and the daily number of new confirmed cases in Brazil have continued to increase rapidly. The total death case in Brazil has reached 560,000, far exceeding that in China, and the effect of epidemic prevention and control is not satisfactory.

**Conclusions:**

Multiple factors, such as meteorological conditions, policies and strategies, and economic conditions, can influence the spread of COVID-19, and therefore, the situation varies greatly from country to country. China and Brazil have chosen different interventions in the fight against COVID-19. The policy measures taken by China are typical containment measures and Brazil has a mitigation strategy. From the perspective of the current situation of the epidemic development in both countries, the cumulative death rate and daily new confirmed cases in Brazil are much higher than those in China, which indicates that the containment strategy is more effective than mitigation strategy in preventing and controlling COVID-19. Fighting the epidemic is a global long-lasting battle, and the two countries should learn from each other with the premise of respecting their national conditions. Countries should deepen cooperation and not let up prematurely.

## Introduction

Novel coronavirus pneumonia is an acute infectious pneumonia, and its pathogen is a new coronavirus previously found in humans, which can constantly adapt to hosts during replication. Novel coronavirus has a higher reproductive capacity than SARS. COVID-19 cases were first detected in Hubei region of China in December 2019. Due to its acute infectiousness, the outbreak spread nationwide and quickly spread around the world [1, 2]. The World Health Organization has assessed that COVID-19 can be classified as a pandemic [3]. On February 7, 2020, the NHC decided to name the "novel coronavirus pneumonia" as "COVID-19" for short. On January 30, 2020, the World Health Organization announced that the COVID-19 epidemic is an internationally concerned public health incident, and its English name is Corona Virus Disease 2019 (COVID-19) on February 11, 2020. As of August 12, 2021, the cumulative number of confirmed cases in the world had exceeded 200 million and the total number of deaths had exceeded 4.33 million. More than a year since the outbreak of the novel coronavirus, this outbreak has posed a huge challenge to the global economy, institutions and management, with devastating effects. The first is the dramatic increase in the number of human deaths around the world. Shutdown, production suspension, seriously affected the economic development of various countries [4]. The emergence of COVID-19 has slowed down global economic development and strongly triggered a global economic crisis [5]. The IMF had predicted that the pandemic would reduce the global economy by four times as much as the 2008–2009 financial crisis. Judging from objective data, since the COVID-19 outbreak, the EU has faced the most serious economic shock since its inception. Since the outbreak of the epidemic, the severe consumption of materials and human resources has put higher demands on the management and governance capacity of governments. The COVID-19 epidemic has had a greater impact on all aspects of China than the SARS epidemic. According to Russian satellite news agency Sputniknews, the Butantan Institute released information that at least 19 coronavirus variants have been detected in Brazil's Sao Paulo state as of June 20, 2021. At present, some regions have seen a rebound of the epidemic, with secondary and tertiary outbreaks, and the future evolution is still unclear. As of August 2021, the epidemic was still present and was aggravated by the emergence of a mutant strain.

The impact of the epidemic is global, and it is a major challenge facing the world. No country can be immune, and until the epidemic ends in one country, the whole world will be at risk [6]. Pandemics and similar pandemics evolve differently in different economic, social, environmental, climatic and institutional Settings [7–9]. Countries have also taken a series of measures in various aspects since the novel coronavirus outbreak. The process of COVID-19 prevention and control also involves the issue of key decisions in management [10, 11]. Governments are making real-time disclosures about the status of COVID-19 infections, tracking and monitoring key populations in real time. The government has paid attention to the voluntary participation of the community in epidemic prevention, and has strictly required people to wear masks when going out. In order to limit population movement, measures such as home quarantine, city lockdown, curfews, and school suspension have been taken [12, 13]. In order to enhance the capacity of the health system and public health, "makeshift hospitals" were built, isolation beds and community isolation facilities were added, vaccine clinical trials were conducted, and emergency response mechanisms were activated. Countries have imposed strict border controls, suspended international flights and visas, and issued a raft of subsidies. Policies and measures adopted by different countries to tackle COVID-19 have their own characteristics, strengths and weaknesses, and the effectiveness of epidemic prevention has also varied significantly.

China, a country with a large population, has taken tough measures to contain the disease. The prevention and control of COVID-19 's epidemic situation was a test of China's governance system and ability [14]. The government has placed the patients under strict quarantine and closely tracked close contacts. The government has strictly followed the three requirements for epidemic prevention and control: controlling the sources of infection, cutting off the routes of transmission and protecting vulnerable groups, and has made it a priority to raise the admission and cure rates and reduce the infection and fatality rates. Brazil, with a population of more than 200 million, is also one of the most populous countries in the world and has been hit hard, at one point being the second-worst country in the world. Brazil was the first South American country to report a confirmed case of Coronavirus Disease 2019 (COVID-19), on February 26, 2020, in Sao Paulo state [15]. The data show that the pandemic in Brazil has grown rapidly since February 25th (date of the first reported case). The Brazilian government has adopted a relatively relaxed mitigation strategy to deal with the outbreak. To mitigate the impact on the economy and to ease the strain on the health system, Brazil has taken a more moderate approach to the outbreak. In this paper, two typical countries, China and Brazil, are selected as the objects of analysis. Based on objective data, the advantages and disadvantages of the epidemic prevention policies of China and Brazil are compared by analyzing the epidemic prevention policies and effects of the two countries.

## Methods

### Data Collection

This study obtained official data on some indicators of COVID-19 in both countries from the official website of the National Health Commission, PRC, the website of the World Health Organization, the website of the Ministry of Health of Brazil and the Novel Coronavirus Resource Centre of Johns Hopkins University. Collect information on specific COVID-19 response measures in both countries from their government websites.

### Statistical Analysis

The effectiveness of the epidemic prevention policies of the two countries was evaluated based on the cumulative number of confirmed cases, daily new confirmed cases, cumulative number of deaths and daily new deaths of the two countries as the main indicators. The study took the policies and strategies adopted by the two countries as independent variables and the epidemic data of the two countries as dependent variables for data analysis.

## Results

### Major COVID-19 Prevention and Control Policies in Both Countries

China was one of the first countries to report COVID-19 and be affected by it. As it was during the Spring Festival travel rush, the virus spread widely in a short time, which brought great difficulties to the government's control and tracking in the later period [16]. With the further spread and impact of COVID-19, the government responded immediately and focused all efforts on epidemic prevention and control. On January 20, 2020, COVID-19 was included in the National Class B infectious diseases, and the prevention and control measures of Class A infectious diseases were taken, as well as the management of the disease Law and the Health and Quarantine Law. Drawing on the experience of SARS, China adopted the "lockdown" measure in January. In the past, the Chinese government has also attached importance to the issue of health equity, so that every citizen can enjoy timely medical services [17]. On February 3, the government pointed out to further improve and strengthen prevention and control, strictly abide by early discovery, early report, early isolation, and early treatment of "four early principles". During outbreaks, real-time monitoring of transmission and mortality rates in countries is a priority [18]. The government has been disclosing daily data to raise residents' awareness of the crisis and has strictly required the wearing of masks. After January 27, China successfully released a notice about the delayed start of school. The epidemic spread rapidly in China in March and has been basically brought under control since April. Then the epidemic prevention and control became normal. At this stage, the nucleic acid testing capacity has been constantly improved, contacts of confirmed cases have been tracked comprehensively, people in isolation have been monitored in real time, and outbreaks have been given full attention. The government has taken a variety of ways to subsidize and rescue residents that have affected by epidemic. Since the prevention and control measures were normalized, sporadic outbreaks have occurred in some areas. Since the normalization of epidemic prevention and control, China has continued to implement the overall prevention and control strategy of "external defense input, internal defense rebound" [19]. The governments of various localities have also actively respond, the whole people cooperate, and the epidemic prevention and control effect is obvious.

Compared with China, the outbreak of COVID-19 in Brazil emerged later and spread slowly in the early stages. The Brazilian government did not take COVID-19 seriously enough and proactively responded to it in the early days, but also took some proactive measures after the outbreak. In response to the unexpected events, the government activated the Public Health Emergency Operations Centre for the Novel Coronavirus. On March 20, 2020, the government declared a "state of public disaster". In the early stages of the outbreak, the government focused on border and coastal zone management and closed the border completely on March 30, 2020. Later, it issued documents to strictly control the land, sea and air. From early March to May 30, Brazil took various prevention and control measures to limit the gathering of people and take quarantine measures to contain the spread of the epidemic [20]. Since the beginning of June, Brazil has gradually lifted the embargo and resumed its economy, relaxed the isolation policy and gradually resumed work and production. But a second outbreak soon followed, with daily new confirmed cases and new deaths rapidly increasing. The rebound has taken a heavy toll, and the government has tightened controls in high-risk areas, imposing curfews and delaying the opening of schools. On January 28, 2021, the vaccination campaign began across Brazil, and by July and August, vaccination coverage increased significantly. Table 1 shows the main policies of China and Brazil in responding to COVID-19.

**Table 1:** Main policies of China and Brazil in responding to COVID-19

Country	China	Brazil
Overall strategy	Containment strategy	Mitigation strategy
Government emergency response	(1) On December 31, 2019, Wuhan Municipal Health Commission publicly notified the situation of a pneumonia of unknown cause, and the first group of experts from the NHC arrived in Wuhan (2) On January 20, 2020, the National Health Commission (NHC) announced that COVID-19 was included in the Class B infectious diseases stipulated in the Law on the Prevention and Control of Infectious Diseases, and that it would be subject to Class A management. Led by the National Health Commission, a joint prevention and control mechanism involving more than 30 departments was set up (3) On August 6, 2021, Beijing established a more scientific hierarchical response mechanism for emergencies, classifying the city's emergency response into four levels from high to low	(1) On January 30, 2020, the Federal Government issued Decree No. 10.211—Supplementary, reactivating the Interministerial Task Force for National and International Major Public health Emergencies (2) On February 3, 2020, in view of the global novel Coronavirus tension, the Ministry of Health of Brazil raised the risk alert for the Novel Coronavirus pneumonia outbreak in Brazil to level 3 ahead of schedule, making it a national public health emergency (3) On March 15, 2021, The state of Sao Paulo entered the "emergency containment phase"
Community and public health	(1) On January 23, 2020, 30 provinces, autonomous regions and municipalities directly under the central government launched the "Level I response to major public health emergencies", formulated and implemented prevention and control measures for communities, and implemented grid and carpet management (2) On January 27, 2020, The State Council approved the extension of the 2020 Spring Festival holiday to February 2. Since then, many places have issued a notice to delay the resumption of work and school (3) On February 2, 2020, the "four categories of personnel" were divided, and on February 3, the "four early principles" were put forward (4) On April 8, 2021, Yunnan province launched the special action of "Clean Restaurant" and "Management and collection City" (5) On April 9, 2021, Yunnan province suspended cinema screenings (6) On June 2, 2021, the prevention and control headquarters of Guangzhou, Foshan and other cities issued a notice, requiring people leaving Guangdong, Guangzhou and Foshan to have a green health code and a negative nucleic acid test within 72 h. A 48-h negative nucleic acid certificate was required from June 7 (7) In June 2021, animal monitoring in closed and controlled areas began in Guangzhou (8) The yellow code system was first introduced in Guangdong in June 2021 (9) On July 25, 2021, all 93 cultural and entertainment venues in Nanjing were suspended (10) On July 26, 2021, the Social and Community Prevention and Control Team of the Joint COVID-19 Prevention and Control Command in Nanjing, Jiangsu Province issued a notice. All residential areas in Nanjing are under strict access control, and no Courier or takeaway worker was allowed to enter the residential areas	(1) On March 14 2020, Sao Paulo, Rio de Janeiro and Brasilia have announced the closure of schools and cancelled major events indefinitely (2) On April 1, 2020, Brazil began urging people to wear face masks when traveling (3) On April 14, 2020, Brazil's Minister of Health requested a complete home quarantine (4) After April 23, 2020, Rio de Janeiro citizens were required to wear face masks with fines for violators (5) On June 27, 2020, the state of Sao Paulo again extended the period of social isolation until 14 July (6) On March 15, 2021, Brazil entered the "emergency containment phase". A "curfew" was imposed from 20 p.m to 5 a.m and vehicle number restrictions were imposed (7) On March 17, 2021, the inland city of Riberon Preto and the northeastern city of SAN Jose Durio Preto were placed under lockdown (8) Sao Paulo state, the epicenter of the outbreak in Brazil, extended its "state of emergency" until April 11, 2021
Health resources and health systems	(1) On January 24, 2020, the government announced the construction of Huoshenshan Hospital (2) On January 25, 2020, the CPC Central Committee established a leading group and announced the construction of Leishenshan Hospital (3) On January 17, 2021, Jilin province identified 66 designated hospitals and 27 reserve designated hospitals, with 6,881 beds available for COVID-19 treatment, including 709 intensive care beds (4)Comprehensive treatment measures were adopted, one person for one policy, according to the policy (5)On April 25, 2020, the Ministry of Commerce, the General Administration of Customs and the State Administration for Market Regulation jointly issued *The announcement on Further Strengthening The Quality Supervision over The Export of Epidemic Prevention Materials* to further standardize the export order of medical supplies (6) On June 25, 2021, Guangdong province proposed the establishment of 5,000 international health stations with independent Spaces (7) On August 13, 2021, In order to meet people's demand for medical treatment and drug purchase during the epidemic period, Nanjing implemented the policy of outpatient referral exemption	(1) On March 23, 2020, the "makeshift hospital" was built at pacanbo Stadium in Sao Paulo, Brazil (2) On April 11, 2020, the Anninbi Makeshift Hospital in Sao Paulo city began operation, receiving a total of 6,350 patients (3) On May 25, 2020, the Federal Government of Brazil purchased 7.5 million masks weighing about 30 tons from China as supplies for the next step in the fight against the epidemic (4) In March 2021, in response to the current shortage of hospital beds, the Health Department of Sao Paulo city built two hospitals for COVID-19 patients, providing a total of 230 intensive care beds and 310 general care beds
Prevention and control of the school	(1) On January 27, 2020, the Ministry of Education announced the postponement of the 2020 spring semester (2) On May 13, 2020, the Ministry of Education stressed efforts to promote the full resumption of classes in all schools and schools to speed up the restoration of normal education and teaching order (3) On June 18, 2020, Beijing Municipal Department of Education also ordered neighboring primary and secondary schools to suspend classes one after another, and senior three students were required to leave school in advance (4) On November 23, 2020, the epidemic rebounded in some areas. Primary and secondary schools were closed again, and students' winter vacation was brought forward (5) On August 4, 2021, Nanjing postponed work related to the school	(1) On February 27, 2020, Brazil's primary and secondary schools were closed (2) Offline teaching began to resume on August 3, 2020, and students returned to school one after another (3) Classes resumed in the northern state of Amazonas on August 10, 2020 (4) Classes resumed in 16 of the capital cities of Brazil's 27 states on August 2, 2021 and other cities resumed offline classes successively
Border and regional control	(1) On January 23, 2020, Wuhan implemented the decision and deployment of the CPC Central Committee and closed its outbound channel. On January 23, 2020, Wuhan was placed under lockdown (2) On March 28, 2020, China imposed strict border control, restricted entry, implemented a health declaration system for entry-exit personnel, and strictly carried out entry health quarantine. China suspended the entry of foreigners with valid Chinese visas and residence permits (3) On July 23, 2020, the Joint Prevention and Control Mechanism of The State Council issued *The notice on Further Improving the Work related to Quarantine and Medical Observation and Nucleic acid Testing of Inbound Travelers*. Inbound travelers who have completed remote nucleic acid testing, meet the conditions for closed transport management, home isolation and precise community control, may be subject to "7 + 7" and "2 + 1" centralized quarantine medical observation measures on a voluntary basis (4) On December 28, 2020, China took strict measures to prevent inbound flights from coming into China and preventing inbound flights from coming back to China. It also strengthened quarantine work at airports and customs, strictly implemented the "14 + 7" measures, and strengthened the management of inbound passengers (5) On April 9, 2021, hongta District required relevant departments to strengthen the tracking of people with travel history in Ruili and those from Myanmar, Vietnam, Laos and other Southeast Asian countries who entered the customs through land ports after March 14 (6) On July 27, 2021, Nanjing implemented epidemic control measures in transportation	(1) On March 30, 2020, Brazil completely closed its borders (2) On April 28, 2020, Brazil issued document No. 203, restricting the entry of foreign citizens into Brazil by air for 30 days starting from April 28 (3)On May 26, 2020, the Brazilian government announced that it would extend *The ban on foreign nationals entering Brazil* for another 30 days. The ban banned people from all countries from entering Brazil by air, land and sea (4) The city of Salvador, the capital of Bahia state, imposed a lockdown on the baja coast, a popular tourist attraction, from 17:00 on December 31, 2020, while all beach areas in the city were closed for one day on 1 January (5) On December 31, 2020, the Brazilian government tightened containment measures, particularly in coastal areas where crowds are most likely to gather (6) On May 28, 2021, Brazil again issued a joint proclamation restricting the entry of foreign citizens into Brazil by road and other means of land and water transport
Policy of lifting restrictions	(1) On February 17, 2020, the Joint Prevention and control Mechanism of the State Council issued *The guidelines on Scientific and Targeted COVID-19 prevention and control by Region and at different levels* (2) On March 25, 2020, outbound travel restrictions were lifted in areas outside Wuhan (3) On April 8, 2020, Wuhan lifted the outbound traffic control measures, and resumed external traffic in an orderly manner, allowing safe and orderly movement of people leaving Wuhan based on hubei health code "Green code". Wuhan was lifted on April 8, 2020 (4) On February 6, 2021, Yunnan province issued the Notice on Forwarding Transportation Services for Local People during the Spring Festival, lifting restrictions on freight transportation in low-risk areas (5)After March 16, 2021, people from low-risk areas in China and people passing around Beijing do not need to check the nucleic acid certificate (6)From July 3, 2021, bus and subway passengers in Guangzhou only need to check their temperature and no longer need to show their health code	(1) On June 1, 2020, the state of Sao Paulo was gradually lifted (2) From June 11, 2020, shopping malls in Sao Paulo were allowed to open for four hours a day, later extended to six (3) On July 6, 2020, restaurants and bars across Sao Paulo were allowed to open from 11 a.m to 5 p.m (4)The mayor of The Brazilian city of Sao Paulo announced plans to close the city's last makeshift hospital, with plans to close it completely on September 10, 2020 (5) From June 1, 2020, the state of Sao Paulo gradually resumed economic activities and the quarantine measures were gradually relaxed in five stages (6) On July 2, 2020, the government announced the reopening of a restaurant in Leblanc, Rio de Janeiro (7) On July 29, 2020, Brazil announced the lifting of its air travel ban, announcing the lifting of restrictions on "inbound passengers by air" in a bid to revive tourism (8) According to a new regulation by the state government of Sao Paulo, starting August 1, 2021, businesses in the state will be able to operate until midnight, eliminating the 11 pm to 5am "curfew". State parks can return to their normal before the pandemic opening hours
Financial aid and grants	(1) On March 3, 2020, a press conference under the Joint Prevention and control Mechanism of The State Council made it clear that the state supports enterprises that support protection and treatment, supply of materials and resumption of work and production. The government has formulated four preferential tax policies to help enterprises resume work and production (2) In March 2020, civil affairs departments in all provinces and cities set up and opened social assistance service hotlines (3) On July 20, 2021, liwan District of Guangzhou launched rescue subsidies for heating enterprises	(1) On March 26, 2020, Brazil's Economy Minister announced that the stimulus package, coordinated by the Economy Ministry, the public bank and the Central Bank, amounted to 750 billion reais (2) Under the Brazilian government's plan, about 90 billion reais will be spent in 2020 to support poor families, including low-income workers, people with no means of income and people on social care (3) On April 6, 2021, a new round of epidemic emergency rescue gold was issued, and the 4 phase was issued. Benefits were paid to irregular workers and participants in Bolsa Familia schemes (4) On July 5, 2021, the Brazilian Hakka event center vaccine station held the "cold winter warm donation blanket" ceremony and cotton-padded clothes and other supplies were delivered to poor areas of Sao Paulo
Vaccine development and vaccination	(1) On March 16, 2020, clinical trials of the novel Coronavirus vaccine were approved in China (2) On December 15, 2020, the first step was to vaccinate key groups, the second step was to vaccinate key and high-risk groups, and the third step was to vaccinate the general population and other groups (3) On December 31, 2020, under the Joint Prevention and control mechanism of The State Council, sinopsin China biological inactivated COVID-19 vaccine was approved for conditional marketing by the State Food and Drug Administration	(1) On September 12, 2020, the Anvisa approved the resumption of clinical trials of the Oxford vaccine in Brazil (2) On January 18, 2021, Brazil's Ministry of Health officially launched the nationwide distribution of COVID-19 vaccines (3) On January 28, 2021, all of Brazil's 26 states and the federal District, where the capital is located, began vaccination (4)On August 14 and 15, 2021, Sao Paulo City launched a new crown virus vaccination activity, mainly for 18 to 21 years old

### Analysis of the Effect of Epidemic Prevention and Control in China and Brazil

1. China's epidemic prevention and control effect
  At the beginning of the outbreak, the Chinese government mainly took preventive measures. In late January and early February, strict prevention and control policies were implemented and home quarantine notices were issued across the country. Figure 1 shows the trend of daily new cases and daily new deaths in China (The left Y-axis represents the daily number of newly confirmed cases in China, and the right Y-axis represents the daily number of new deaths). As can be seen from the Fig. 1, in the early days of the outbreak, daily new cases remained at a relatively high level. It peaked at 15,152 cases on February 13, 2020, and then gradually decreased and remained at a low level. By April 19, there were only 21 cases. In terms of daily new deaths, the rate of increase was slow until February 1, then increased. The peak was 1,290 on April 18. Since then, the daily number of new deaths has remained at a low level until June 2021, when the trend has increased. China's containment strategy has dramatically reduced the death and morbidity rates from COVID-19 [21]. Since the normalization of epidemic prevention and control, some provinces and cities in China still have outbreaks, with small fluctuations. In April 2021, the epidemic rebounded in Yunnan. High risk areas appeared in many places in Guangzhou in May and June. The epidemic broke out in Nanjing in July and August and spread to many provinces across the country. At present, the epidemic in China is not completely over. It is sporadic on the whole and there is a high risk in some areas.

**Fig. 1:**
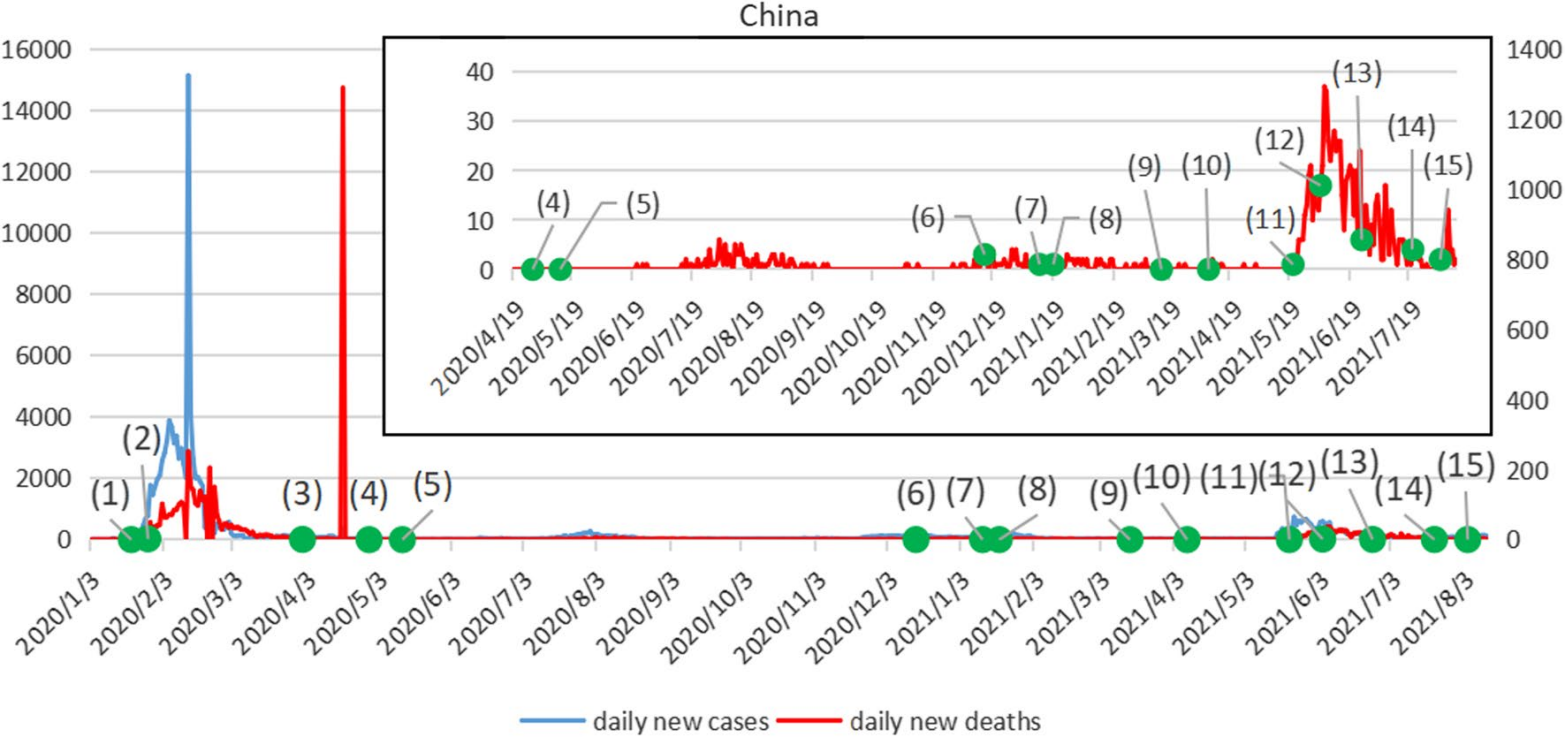
Trends of daily new cases and daily new deaths in China. *Note: *(1)On January 20, 2020, the National Health Commission included COVID-19 as a Class B infectious disease under the Law on the Prevention and Control of Infectious Diseases and implemented a Class A management. (2)On January 27, The State Council approved the extension of the 2020 Spring Festival holiday to Feb 2. (3)On April 1, the government conducted nucleic acid sampling tests on all passengers entering China through air, water and land ports, and took measures of medical treatment or centralized isolation by category. (4)On April 29, the epidemic was sporadic in China, and imported cases were basically under control. The state declared that the epidemic prevention and control had entered a phase of normalization. (5)On May 13, the government began to promote the full resumption of classes in all schools. (6)On December 15, the first step to vaccinate targeted groups began. (7)On January 12, 2021, shijiazhuang city, Xingtai City, Langfang city implemented the closed management and the most stringent "four must" measures. (8)on January 19, the commerce Ministry received its first aid package. (9)On March 15, primary and secondary schools and kindergartens in Beijing took regular measures to prevent the epidemic. (10)On April 8, Market Supervision Bureau of Yunnan Province continued to strengthen the promotion and application of "Yunzhisu" platform, and implemented the "111" working mechanism. (11)On May 21, guangdong province conducted a high-quality epidemiological survey. (12)On June 4, Guangzhou City launched a large investigation of nucleic acid detection in the city. (13)On June 25, Guangzhou proposed the establishment of 5000 international health stations with independent space. (14)On July 21, Nanjing COVID-19 Prevention and Control Headquarters issued the 《Notice of Nanjing COVID-19 Prevention and Control Headquarters on Further Strengthening COVID-19 Prevention and Control work 》. (15)On August 4, Nanjing postponed work related to the school
2. Brazil's epidemic prevention and control effect
  Figure 2 shows trends in daily new cases and daily new deaths in Brazil (The left Y-axis represents the daily number of newly confirmed cases in Brazil, and the right Y-axis represents the daily number of new deaths). The epidemic in Brazil can be divided into two stages. First, during the first wave of the epidemic, the first confirmed case in Brazil was on February 25, 2020, and the daily new confirmed cases remained at a low level for some time thereafter. The first death occurred on March 17, and by March 18, 2020, the number of newly confirmed cases per day had exceeded 100. Since 1 April, the number of newly confirmed cases per day has exceeded 1,000. On April 5, 2020, Brazil surpassed 10,000 cumulative COVID-19 cases, making it the worst-affected country in South America. Since June 2020, Brazil has gradually relaxed its control and gradually imposed contact restrictions, which has led to the outbreak not being effectively controlled. The first peak occurred in July and August, after which it decreased, but remained at a high level. Starting in November, the epidemic rebounded with a second wave of outbreaks. Brazil once ranked third in cumulative cases and second in new cases. Since 2021, The epidemic has been rapidly spreading in Brazil, with a high daily number of new cases and daily new deaths. The number of people who have died from COVID-19 in the country has topped half a million in just over a year. It took 149 days, 152 days, 76 days and 36 days respectively to reach 100,000, 200,000, 300,000 and 400,000 deaths. The Brazilian epidemic is characterized by another peak on top of the first. The epidemic in Brazil has gradually expanded and remained at a high level for a long time, presenting a grim situation.

**Fig. 2:**
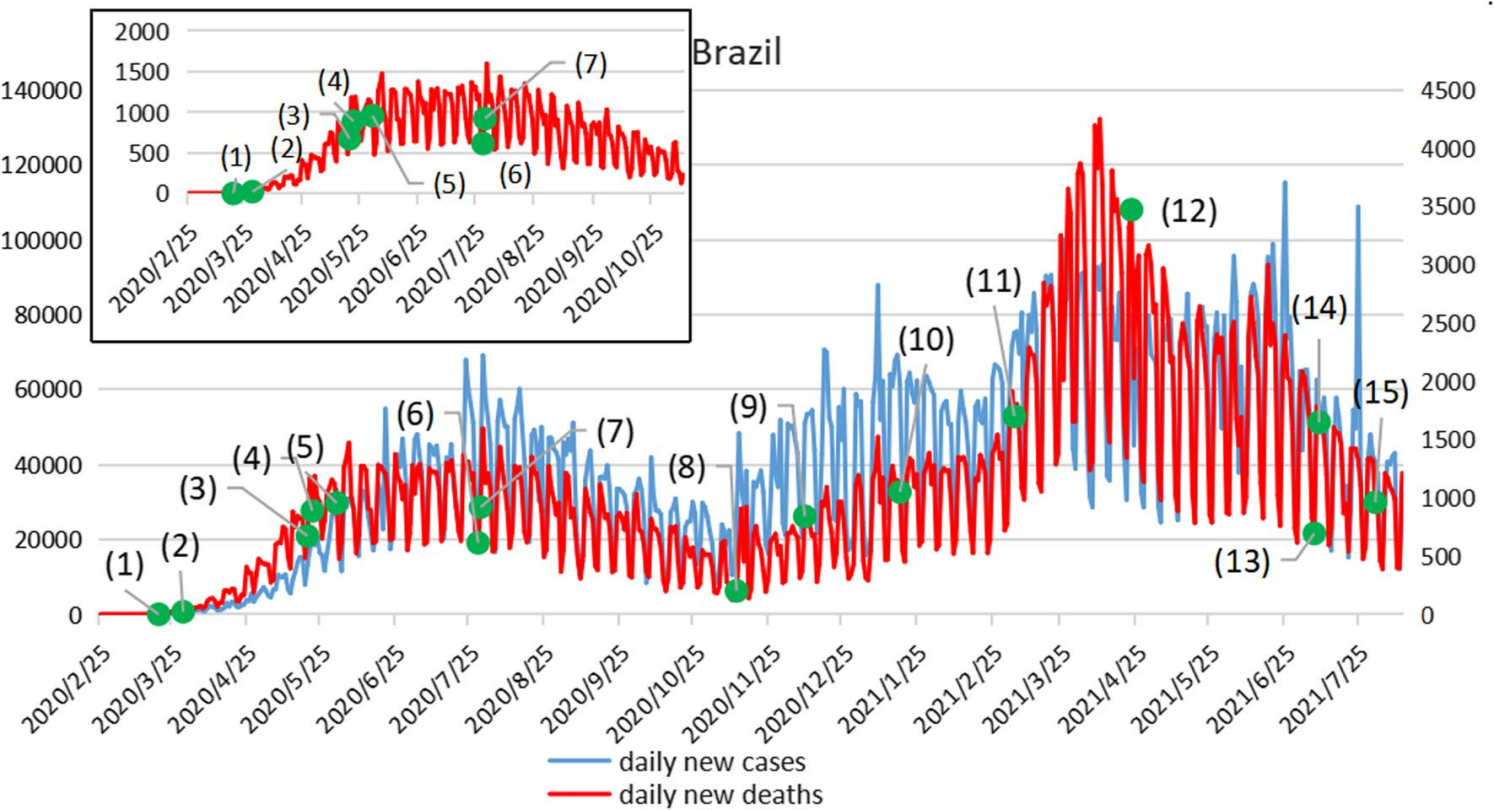
Trends of daily new cases and daily new deaths in Brazil. Note: (1)On March 20, 2020, Brazil's federal government declared a state of public disaster. (2)On March 30, Brazil closed the border completely. (3)On May 20, Brazil's parliament approved a law that makes wearing face masks mandatory in public, with fines for failing to comply. (4)On May 22, Brazil issued document No. 255 restricting entry of foreign citizens through land, sea and air ports for 30 days. (5)On June 1, Sao Paulo state was gradually unsealed. (6)On July 29, Brazil announced the lifting of its air travel ban, announcing the lifting of restrictions on passengers entering the country by air. (7)From July 30 to August 3, the Butantan Institute of Brazil provided drive-in Novel Coronavirus nucleic acid tests to the public in a shopping mall car park in Sao Paulo. (8)On November 12, a joint announcement was issued on measures to restrict the entry of foreign citizens into Brazil through land and sea ports within 30 days from November 12. (9)On December 10, Brazil's Ministry of Education approved a resolution allowing schools across the country to maintain online delivery. (10)From January 18 to 19, 2021, all of Brazil's 26 states and the federal District, where the capital is located, began vaccination. (11)On March 6, Sao Paulo entered the "red phase," the highest phase of the epidemic. (12)On April 23, the Brazilian government unveiled the 2021 budget bill signed by President Jair Bolsonaro on April 22 to tackle the public crisis through spending cuts. (13)On July 7, Sao Paulo State Governor Joao Doria announced the resumption of offline courses at public and private universities. (14)On July 9, Sao Paulo state began to relax quarantine measures and extend business hours. (15)On August 1, the Brazilian state of Sao Paulo has lifted its curfew
3. Comparative analysis of the epidemic situation in the two countries
  Figure 3 shows the trend of total death cases in China and Brazil. As can be seen from the figure, the time of death in China was relatively early. On January 28, 2020, the total death cases exceeded 100, and on February 10, 2020, it exceeded 1,000, showing a rapid growth in the early stage. Thereafter, the daily new death case was mostly maintained in the number of digits, and the number of death cases was in a slow growth. On August 12, 2021, the total death case in China was 5,664. The death case in Brazil was later than that in China, and the total increase rate of death was at a relatively low level until April 2020. The total death cases exceeded 100 on March 30, 2020, and surpassed 1,000 in just a dozen days. It began to grow rapidly after April and began to overtake China on April 30. At present, the total number of deaths in Brazil is still in a state of rapid growth. In terms of total deaths, there is a significant gap between the two countries in the effectiveness of epidemic prevention and control.

**Fig. 3:**
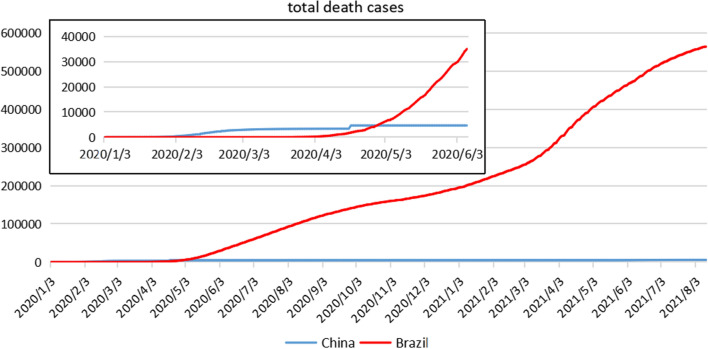
Trend chart of total death cases in China and Brazil

## Discussion

### Complex Relationships Between the COVID-19 Pandemic and Socio-economic and Meteorological Conditions

The Novel Coronavirus is highly uncertain and complex in both space and time. Effective public governance can support health systems to be prepared in a pandemic environment [22]. Environmental conditions were found to be a factor in the spread of the virus across geographical boundaries [23]. The social distancing and preventive measures instituted across countries have a link with spread containment whereas spread containment is associated with meteorological factors[24]. For example, both atmospheric parameters and air quality are correlated with COVID-19 outbreaks [25, 26].

**Fig. 4:**
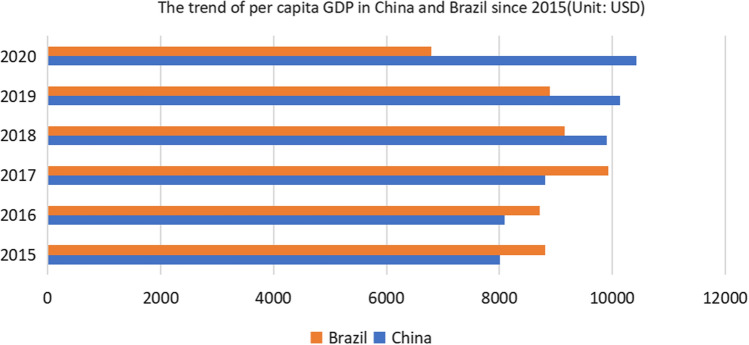
The trend of per capita GDP in China and Brazil since 2015

1. Novel Coronavirus and Aerosols
  Aerosol is a gaseous dispersion system composed of solid or liquid particles suspended in a gaseous medium. Coughing, sneezing, and even breathing produce aerosols. Small droplets in a room without air flow can hover for seconds to minutes, but with the right air flow, these droplets move and spread faster. Studies have shown that COVID-19 can be transmitted by aerosols. COVID-19 can be transmitted by aerosols in confined Spaces, but rarely in open environments.
2. Novel Coronavirus and Temperature
  A study has found that temperature is an environmental driver of the COVID-19 outbreak in China. Low temperatures and high temperatures may help reduce the incidence of COVID-19. Thus, Brazil's perennial hot weather conditions may help contain the spread of COVID-19. In another study of 122 cities in China, the average temperature was found to have a positive linear relationship with the number of COVID-19 cases when the temperature was below 3 degrees Celsius. There is no evidence that the number of COVID-19 cases will decline as the weather warms [27].
3. Novel Coronavirus and Humidity
  Many studies around the world have documented that humidity plays a critical role in COVID-19 morbidity and mortality. In a study of Bangladesh, high temperatures and relative humidity were found to slow the spread of COVID-19 [28]. A high correlation between novel coronavirus transmission time and absolute humidity has been found in a comparison of China, the UK, Germany and Japan [29]. China and Brazil have their own characteristics in terms of precipitation and humidity, which may also contribute to the difference in the epidemic situation.
4. Novel Coronavirus and Wind Speed
  In a study of Italy, it was found that the lower the wind speed and the more severe the air pollution, the higher the number of novel Coronavirum-related infections [30]. Strong wind speeds in China's coastal and northwestern regions may help contain the spread of the pandemic. Despite strong winds along the coast of Brazil, there has been a decrease in winds inland, which may contribute to the spread of COVID-19 in some areas.
  Other indicators of climate and environmental pollution have also been linked to the spread of COVID-19 [31–33]. So one of the current problems in environmental science is to explain how air pollution is affecting the COVID-19 pandemic [34, 35]. In Rio de Janeiro, Brazil, for example, weather has played a key role in the spread of COVID-19. Solar radiation is the most critical weather factor and has a significant and strong correlation with the onset of COVID-19 [36]. Some research results have shown that long-term exposure to certain air pollutants can cause more severe COVID-19 and delay recovery for patients [37, 38]. Policies such as lockdowns and quarantines during COVID-19 have also had an impact on air quality [39]. At the same time, the impact of meteorological factors on COVID-19 is inconsistent and contradictory, because the development of COVID-19 is related to multiple factors, and a single meteorological factor cannot fully explain the current situation of the epidemic. In addition to meteorological factors, COVID-19 also interacts with a country's economic growth [40]. Figure 4 shows the trend of GDP per capita in China and Brazil since 2015. On the one hand, a country's economic situation has restricted epidemic prevention and control, and on the other hand, the COVID-19 pandemic has seriously affected a country's economic development. Studies have found that in many countries, the COVID-19 pandemic has significant ethnic differences, with minority populations disproportionately infected [41, 42]. Table 2 shows the major factors influencing the COVID-19 outbreak in the two countries.

**Table 2:** Major factors influencing the COVID-19 outbreak in the two countries

Country	China	Brazil
Per capital GDP	China's per capita GDP in 2020 is $10,434	Brazil's GDP per capita in 2020 is $6,796
Policy strategy	Containment strategy	Mitigation strategy
Variants	Variants have been found in China	Variants have been found in Brazil
Vaccination	Since the outbreak, the Chinese government has actively developed vaccines and started vaccination early	Brazil is late to mass vaccination
Main climatic types	Subtropical monsoon climate, temperate monsoon climate and temperate continental climate	Tropical rainforest climate and savanna climate
Temperature	Summer is hot and winter is cold and dry	High temperature all the year round
Wind speed	The wind speed is higher in qinghai-Tibet Plateau, northwest, southeast coastal areas and islands, and lower in inland areas	Brazil has excellent wind conditions and wind power is one of the country's most important sources of energy
Humidness	The precipitation and humidity are large in the southeast coastal areas, while the precipitation and humidity are small in the inland and northwest areas	Brazil has a lot of precipitation, high humidity, some parts of the dry and wet seasons
Ethnicity	China is a unified multi-ethnic country, and the Han population occupies a dominant position in the ethnic structure of China's population. Besides the Han, There are 55 ethnic minorities in China	There are mainly Indians, black and white, and mixed race people in Brazil. Due to historical reasons, the ethnic composition of Brazil's population is very complex, and cultural differences are marked

The COVID-19 pandemic posed challenges for healthcare systems and political leaders across the globe [43]. China and Brazil have differences in their systems of government, healthcare and attitudes towards the epidemic. The two countries have adopted containment and mitigation strategies according to their national conditions. As can be seen from the above analysis, the epidemic prevention effects of the two countries are also significantly different. China has brought the rapid spread of the epidemic under control in just a few months, with daily new cases and deaths returning to low levels. Although some regions have seen a rebound, secondary outbreaks have been avoided as a whole. Brazil's response has been less than satisfactory. Some of Brazil's early policies did have a certain prevention and control effect, but the relaxation of the later period led to a serious worsening of the epidemic in Brazil. The daily number of newly cases and the total number of confirmed cases are soaring. Brazil was once the second worst country in the world and has not yet been well controlled.

One of the strategies of defense against infectious diseases (e.g., COVID-19) is the vaccinations that decrease the numbers of infected individuals and deaths. In this context, the optimal level of vaccination for COVID-19 is a basic point to control this pandemic crisis in society. Findings reveal that the average level of administering about 80 doses of vaccines per 100 inhabitants between countries can sustain a reduction of confirmed cases and number of deaths [44]. Therefore, improving public confidence in vaccination is also an important measure to control the outbreak [45]. According to statistics, as of July 16, 2021, there were a total of 135 candidate vaccines and a total of 391 vaccines in trials, including 36 vaccines in phase I clinical trials, 54 vaccines in phase II clinical trials, 40 vaccines in phase III clinical trials, and 20 approved for use. At present, the world has entered the phase of vaccination, countries should be universal vaccination as soon as possible. Meanwhile, non-drug prevention and control measures should not be relaxed [46]. Israel, a model vaccination country, has an 85% adult vaccination rate, far ahead of any other country in the world. COVID-19 dramatically influenced mortality worldwide, in Italy as well, one of the first European countries to experience the COVID-19 epidemic [47]. The number of new infections in Israel dropped from an average of more than 10,000 a day in January 2021 to more than 100 in April. Later, the Israeli government decided to lift the ban completely, and by June, masks were no longer required. But the Delta virus quickly took advantage and spread very quickly. By July 2021, when cases spiked to more than 1,000 a day, masks were reactivated and contacts of confirmed cases were quarantined for 14 days. By August 12, 2021, the daily number of newly cases reached 6,022, and the total number of confirmed cases reached 919,602, indicating a significant rebound of the epidemic. For infectious diseases, prevention and control is more important than treatment and improve the whole people. Awareness of self-protection, overall planning and coordination of epidemic isolation is very important [48]. Therefore, although vaccination plays an important role in epidemic prevention and control, it is not enough to rely on vaccination alone to prevent and control the epidemic, and non-drug interventions are still the top priority in current work.

### Assessment of Containment Strategies

China is following a classic containment strategy. Rapid response is critical in the early stages of an infectious disease outbreak [49]. In the early stages of the epidemic, the government's main task was to control and screen people. Later, the government realized the seriousness of the epidemic and the threat to people's lives, and adopted a strict containment strategy. The government launched a comprehensive screening of travelers and continued to test for cases. Quarantine measures can effectively reduce the spread of infectious viruses [50]. The government also suppressed the spread of the outbreak by tracking and isolating suspected cases and close contacts. For the management of community residents, comprehensive screening, real-time monitoring and wearing masks are required. China has given full play to the key and core role of the community in epidemic prevention and control. The community has strictly adhered to the state's quarantine policy and has developed its own response to the epidemic. In terms of the health system, in order to relieve the pressure on the medical system and enhance the capacity of the medical system, the government built more wards and hospitals and actively organized personnel to develop vaccines. China has controlled the rapid spread of the epidemic in just a few months and the epidemic has been effectively brought under control. It turns out that the most effective way to prevent and control COVID-19 is early detection, diagnosis, isolation and treatment [51]. On August 12, 2021, the total number of confirmed cases in China was only 122,058, with 98 newly confirmed cases. By August 12, 2021, Germany, which also adopted the containment strategy, had a cumulative death case of 91,834, with 44,887 confirmed cases and 17 new deaths, and the epidemic was effectively prevented and controlled.

### Assessment of Mitigation Strategies

Mitigation strategies aim to keep the number of infections low through modest control measures, but could overwhelm health service capacity if COVID-19 infections increase [52]. Brazil is an example of a mitigation strategy. In the early stages of the COVID-19 outbreak, Brazil only admitted, isolated and tested severe patients, but did not pay enough attention to mild patients, who were advised to stay at home for self-observation. As for the control of residents, the government has taken various measures to limit the gathering of people, including suspending classes and canceling large-scale events indefinitely. People were urged to wear masks in public places and implement home quarantine policies. However, Brazil's president backed only elderly people staying at home, citing concerns about jobs. Later, due to the aggravation of the epidemic, many cities adopted a total ban and lockdown measures, or extended the quarantine period. The COVID-19 epidemic was a test of Brazil's unified health care system [53]. COVID-19 is causing the collapse of Brazil's national health service [54]. In terms of health systems, on March 23, Brazil built a "makeshift hospital" inside the Pacanbo Stadium in Sao Paulo. The construction of square cabin hospital was a new concept to deal with sudden health incidents [55]. Sao Paulo's Annimbi Makeshift Hospital opened on April 11 and has treated 6,350 patients. Since June 1, the state of Sao Paulo has gradually resumed economic activities, and five colors of red, orange, yellow, green and blue have been used to classify and control the epidemic. Mitigation strategies can slow the spread of the epidemic to some extent and reduce the pressure on the medical system, but they cannot completely control the epidemic, which is also an important factor in Brazil's second resurgence of the epidemic. As of August 12, 2021, the total number of confirmed cases in Brazil has reached 2,0212,642, with 34,885 new confirmed cases on that day. On August 12, 2021, the total number of confirmed cases and total death case in the United States, which also adopted mitigation strategies, reached nearly 36 million and 613,647. The epidemic has not been well controlled in the two countries.

## Conclusions

The epidemic has caused a severe impact on the medical and economic aspects of the world, which should draw the attention of all parties. This study makes a comparative analysis of typical containment and mitigation strategies in China and Brazil. Based on the situation of epidemic prevention in the two countries, the evaluation of the two epidemic prevention policies shows that China, which implemented the containment strategy, has controlled the epidemic in a relatively short period of time. Brazil, which implemented a mitigation strategy, relaxed its guard after the epidemic eased slightly, causing the epidemic to rebound. The epidemic has caused heavy human losses, and Brazil's prevention and control effects are not satisfactory. China, as a country with a large population, is more difficult to prevent and control, but the epidemic prevention and control effect is more obvious. Therefore, from the perspective of the current prevention and control effects in both countries, containment strategies are better than mitigation strategies in the prevention and control of COVID-19.

In addition to non-pharmaceutical interventions, reducing vaccine hesitancy and implementing an effective vaccination programme are also important measures to contain the COVID-19 pandemic. The evolution of the COVID-19 pandemic has been linked to multiple factors, for example, a variety of socio-economic and environmental factors have played an important role in explaining evolutionary dynamics over time and space during the COVID-19 pandemic. However, data sources can only capture some aspects of the ongoing outbreak dynamics. The results here are mainly based on the policy strategies of the two countries, and future investigations must expand the research scope and increase the factors to be considered.

## Data Availability

All data generated or analysed during this study are included in this published article.
